# A new noninvasive technique for estimating hepatic triglyceride: will liver biopsy become redundant in diagnosing non-alcoholic fatty liver disease?

**DOI:** 10.1186/s12916-014-0152-z

**Published:** 2014-08-26

**Authors:** Bark Betzel, Joost PH Drenth

**Affiliations:** Department of General Surgery, Rijnstate Hospital, Wagnerlaan 55, Arnhem, 6815 AD the Netherlands; Department of Gastroenterology and Hepatology, Radboud University Medical Center, PO Box 9101, Nijmegen, 6500 HB the Netherlands

**Keywords:** Bariatric surgery, Hepatic fat concentration, Magnetic resonance imaging, Metabolic syndrome, Non-alcoholic fatty liver disease, Non-alcoholic steatohepatitis

## Abstract

Obesity and metabolic syndrome are healthcare problems that continue to rise in frequency worldwide. Both phenotypes are a strong predictor for development of liver steatosis in the context of non-alcoholic fatty liver disease or non-alcoholic steatohepatitis. Ultrasound may detect steatosis, but its sensitivity is limited and liver biopsy is still considered to be the gold standard. Less invasive techniques that accurately quantify liver steatosis are warranted. Jiménez-Agüero and colleagues propose that multi-echo magnetic resonance imaging might be such a diagnostic tool. They validated multi-echo magnetic resonance imaging with measured hepatic triglyceride concentration. Their results show that this innovative technique measures the grade of steatosis in different clinical situations. Therefore, multi-echo magnetic resonance imaging might be considered for monitoring liver steatosis as an intermediate endpoint. Wide clinical applicability is limited though, as it does not allow differentiation between non-alcoholic fatty liver disease and non-alcoholic steatohepatitis.

## Background

The incidence of obesity is increasing worldwide at an alarmingly fast pace, mainly because of a combination of cultural, social, and economic factors that affect lifestyle. Currently, one out of every three Americans is considered to be obese. Obesity is the main driver for the onset of metabolic syndrome, which is a group of metabolic abnormalities that share the cardiovascular risk factors hypertension, dyslipidemia, and hyperglycemia. Presence of metabolic syndrome including obesity is a strong predictor for hepatic steatosis, defined as lipid deposition in hepatocytes. Its phenotype ranges from non-alcohol fatty liver disease (NAFLD), to non-alcoholic steatohepatitis (NASH - fatty changes with inflammation and hepatocellular injury or fibrosis), to advanced fibrosis and cirrhosis.

Similar to obesity, NAFLD has reached epidemic proportions with a prevalence of 20% to 30% in western populations [1]. Because NAFLD constitutes a risk factor for NASH, it is to be expected that the prevalence of complicated liver disease in patients with obesity and metabolic syndrome will rise. As such, prevention of metabolic syndrome is a therapeutic target and lifestyle changes for patients at risk should be encouraged.

The question then arises, how to detect liver steatosis [2]? Ultrasound is by far the most frequently used modality to detect liver steatosis. Fatty filtration of the liver produces a diffuse increase in echogenicity (a bright liver) and blurring of vascular margins and the diaphragm. Ultrasound, however, has some inherent disadvantages, such as a limited sensitivity to detect steatosis when less than 30% of hepatocytes contains fat, and its accuracy is compromised in patients who are obese. In addition, ultrasound cannot identify relevant liver disease in the context of NASH, such as steatohepatitis or fibrosis.

The gold standard for diagnosis of NAFLD and to differentiate it from NASH is liver biopsy. A liver biopsy should be considered in patients with liver steatosis on imaging who are at increased risk of steatohepatitis and advanced fibrosis, such as patients with competing etiologies for hepatic steatosis (for example, diabetes mellitus) and with co-existing liver disease. However, the invasive nature of liver biopsy with its risk for major complications, such as bleeding in approximately 1% of cases [3], precludes widespread use. Therefore, an accurate, easy to conduct, and less invasive technique is needed in diagnosing NAFLD [4]. Both computed tomography and magnetic resonance imaging (MRI) are more sensitive modalities for quantifying steatosis. However, none of these imaging techniques have sufficient sensitivity and specificity to stage the disease and cannot distinguish between NAFLD and NASH [5].

Jiménez-Agüero and colleagues [6] performed a rigorously designed and well-executed prospective study in 129 patients where hepatic lipid concentration was determined by three different methods. They performed histopathological examination of liver biopsies, measured hepatic triglyceride concentration from liver biopsy specimen (Folch method), and established liver fat fraction using multi-echo MRI. Multi-echo MRI was performed within 24 hours before collection of the biopsy, so no changes in hepatic fat content could occur due to the time interval. To improve diagnostic accuracy, they developed an equation that took into account the multi-echo MRI data, which was then validated in an additional cohort of 31 patients. After validation of the multi-echo MRI, this tool was tested to see if it could detect changes in liver fat content over time in obese patients. From the original cohort of 97 obese patients, 86 underwent bariatric surgery while 11 were treated by partial liver resection. In total, 66 (56 and 11) patients received a second multi-echo MRI one year after surgery.

## Applicability

Jiménez-Agüero and colleagues [6] validated a noninvasive tool (multi-echo MRI) with a biochemical quantification of hepatic triglyceride concentration. The key finding of the study is that accurate grading of steatosis with help of multi-echo MRI becomes possible. There are clear advantages of a noninvasive technique, especially when it comes to follow-up of patients with NAFLD undergoing treatment. In their hands, multi-echo MRI was able to predict the concentration of liver fat and demonstrated the improvement of steatosis after bariatric surgery. By contrast, in obese patients who underwent partial liver resection, hepatic steatosis remained unchanged. As such, multi-echo MRI allows monitoring of changes in hepatic fat content in different clinical situations.

Unfortunately, the applicability of this study in clinical practice stops there; it allows the differentiation between grades of steatosis. The technique does not differentiate between steatosis, steatohepatitis, and fibrosis. The ability to perform the latter is essential because the presence of steatohepatitis and fibrosis affects prognosis and influences disease management [7].

## Bariatric surgery

**Figure 1:**
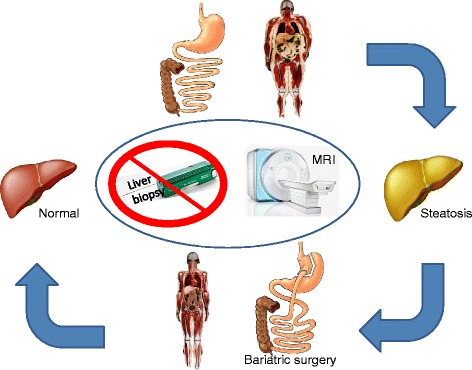
**Bariatric surgery leads to a decrease of liver steatosis, which can be detected by multi-echo magnetic resonance imaging.**

The researchers took bariatric surgery as a model. Although patients eligible for bariatric surgery have a substantial risk for metabolic syndrome, it should be noticed that not all these patients develop NAFLD: 11% of their obese patients did not have steatosis. The data of Jiménez-Agüero *et al*. [6] suggests that bariatric surgery reduces the liver fat fraction as measured by multi-echo MRI (Figure 1). Although bariatric surgery has superior effects on obesity and diabetes mellitus compared to conservative treatment [8], findings regarding beneficial effects on NAFLD and NASH are inconclusive [9]. Indeed, no randomized controlled trials have examined the effect of bariatric surgery on other components of metabolic syndrome, such as NAFLD or NASH. However, some anecdotal reports (such as the paper of Jiménez-Agüero *et al*. [6]) suggest regression and/or histological improvement of NAFLD or NASH after weight loss induced by bariatric surgery [10]. A rigorous clinical trial suggested that weight loss achieved through intensive lifestyle changes leads to improvement of histological signs of NASH [11].

## Future perspectives

Multi-echo MRI is an attractive noninvasive technique to measure the grade of steatosis. However, as long as the grade of hepatic fat has no direct effect on liver disease, in contrast to NASH, multi-echo MRI at best measures an intermediate endpoint. Histopathological assessment of hepatitis and fibrosis, by means of a biopsy, remains the gold standard to differentiate between NAFLD and NASH. Therefore the use of multi-echo MRI is probably restricted to clinical research. On a different note, we encourage the development of new noninvasive techniques to grade steatosis because they can be of great value in patients with NASH. There, these techniques can play a role in monitoring the reduction of steatosis through lifestyle changes. In such cases, steatosis is a worthwhile intermediate endpoint that is associated with improved histological lesions that occur in NASH, such as hepatitis, hepatocellular ballooning and fibrosis.

## Abbreviations

MRI: magnetic resonance imaging
NAFLD: non-alcoholic fatty liver disease
NASH: non-alcoholic steatohepatitis
